# Reduced incidence and economic cost of hardware removal after ankle fracture surgery: a 20-year nationwide registry study

**DOI:** 10.1080/17453674.2020.1733749

**Published:** 2020-02-28

**Authors:** Nikke Partio, Tuomas T Huttunen, Heikki M Mäenpää, Ville M Mattila

**Affiliations:** a Department of Orthopaedics and Traumatology, Tampere University Hospital, Tampere;; b Department of Emergency, Anesthesia and Pain Medicine, Tampere University Hospital;; c Faculty of Medicine and Health Technology, Tampere University, Finland

## Abstract

Background and purpose — Open reduction and internal fixation (ORIF) is a treatment method for unstable ankle fractures. During recent years, scientific evidence has shed light on surgical indications as well as on hardware removal. We assessed the incidence and trends of hardware removal procedures following ORIF of ankle fractures.

Patients and methods — The study covered all patients 18 years of age and older who had an ankle fracture treated with ORIF in Finland between the years 1997 and 2016. Patient data were obtained from the Finnish National Hospital Discharge Register.

Results — 68,865 patients had an ankle fracture treated with ORIF in Finland during the 20-year study period between 1997 and 2016. A hardware removal procedure was performed on 27% of patients (n = 18,648). The incidence of hardware removal procedures after ankle fracture decreased from 31 (95% CI 29–32) per 100,000 person-years in the highest year 2001 (n = 1,247) to 13 (CI 12–14) per 100,000 person-years in 2016 (n = 593). Moreover, the proportion and number of removal operations performed within the first 3 months also decreased. The costs of removal procedures decreased from approximately €994,000 in 2001 to €472,600 in 2016.

Interpretation — Removal of hardware after ankle surgery (ORIF) is a common operation with substantial costs. However, the incidence and cost of removals decreased during the study period, with a particular decrease in hardware removal operations within 3 months.

It is estimated that approximately 40% of all ankle fractures require surgical management, most commonly open reduction and internal fixation (ORIF) (Jensen et al. 1998). According to Kannus et al. (2016), an earlier increasing trend in the incidence of ankle fractures in Finland has steadied.

Ankle fractures are associated with high costs related not only to the operation and subsequent hospitalization, but also to the duration of occupational disability (Stull et al. 2017). To reduce the costs of occupational disability, an early return to previous activities and the avoidance of secondary operations is crucial. In a recent study, Fenelon et al. (2019) found that 13% of patients who had had ankle fracture surgery in Ireland underwent hardware removal. The most common reason was planned removal (6%) followed by symptomatic hardware (6%), and infection (0.5%). The reasons for hardware removal include pain and soft tissue irritation, deep late infection, metal allergy or toxicity, hardware migration, metal failure, and secondary fracture (Bostman and Pihlajamaki 1996). The hardware removal rates reported by previous studies have varied between 12% and 80% (Richards et al. 1992, Sanderson et al. 1992, Bostman and Pihlajamaki 1996).

While the removal of hardware after ankle fracture surgery is often a straightforward procedure, complication rates are still as high as 10–20% (Sanderson et al. 1992, Kasai et al. 2019). Patient satisfaction and symptomatic relief following ankle fracture surgery is also controversial (Jamil et al. 2008, Williams et al. 2012). Postoperative complications include infections, impaired wound healing, refractures, tissue and nerve damage, postoperative bleeding, and incomplete removal (Sanderson et al. 1992).

We determined the incidence and trends in Finland of ankle fracture surgery and hardware removal after ORIF of ankle fractures on a national level. Additionally, we estimated the costs and economic burden of the removal and surgery itself.

## Patients and methods

### Study design and data sources

The Finnish National Hospital Discharge Register (FNHDR) was founded in 1967. It provides data on age, sex, domicile of the subject, duration of hospital stays, primary and secondary diagnosis, and surgical procedures performed during the hospital stay. The data collected by the FNHDR are mandatory for all hospitals, including private, public, and other institutions. The validity of the FNHDR has been proven to be excellent regarding both the coverage and accuracy of the database.

In 1996, the Nordic Medico-Statistical Committee (NOMESCO) published the first printed edition of the NOMESCO Classification of Surgical Procedures (NCSP) followed by the Finnish translation (NCSP-F) in 1997.

### Study population

We included all adult patients (≥ 18 years) with a diagnosis of ankle fracture (ICD-10 code) and who underwent ankle fracture surgery (ORIF) between January 1, 1997 and December 31, 2016.

The procedural codes (according to the Finnish version of the NOMESCO classification) for the ankle fracture included NHJ10 (internal fixation of fracture of ankle using plate, wire, rod, cerclage, or pin) and NHU20 (hardware removal). The primary outcome was the incidence of operative treatment of ankle fractures and hardware removal. Since the FNHDR does not include laterality of the operation, only the patient’s 1st ankle fracture ORIF operation performed during the study period was included in the analysis.

A secondary outcome was the cost of hardware removal as determined by diagnosis-related group-based (DRG-based) hospital payment system pricing. In Finland as well as in most Organization for Economic Co-operation and Development (OECD) countries, DRG-based hospital payment systems are being used. The basic idea of DRG-based hospital payment systems is that all patients treated by a hospital are classified into a limited number of DRGs that are supposed to be clinically meaningful and relatively homogeneous in their patterns of resource consumption.

### Statistics

The trends for operatively treated ankle fracture and hardware removal (per 100,000 person-years) were based on the entire adult population of Finland rather than cohort or sample-based estimates. Mid-year population size was estimated by taking the geometric mean of year-end population sizes of consecutive years. Incidence density rate and operations total rate are presented with the 95% confidence interval (CI). Data were analyzed with R 3.5.3 (R Foundation for Statistical Computing, Vienna, Austria).

### Ethics, registration, funding, and potential conflicts of interest

The study was approved by Finland: National Institute of Health and Welfare (THL): THL/89/5.05.00/2012. The study was funded by Finland’s government research and development foundation. Funding sources were not involved in study design, collection, analysis, interpretation, or completion. The authors declare no conflicts of interest regarding this study.

## Results

68,865 adult patients (51% women) had an ankle fracture surgically treated with ORIF in Finland during the 20-year study period between 1997 and 2016. The mean age at the time of the 1st surgery in 1997 was 52 years in women and 44 years in men. In 2016, the corresponding ages were 57 years in women and 47 years in men. The total incidence of ankle fracture surgery was 81 (CI 78–83) per 100,000 person-years in 1997 (3,218 operations) and 74 (CI 71–76) per 100,000 person-years in 2016 (3,276 operations) (Figure 1). In men, the incidence was 87 (CI 83–91) per 100,000 person-years in 1997 and 72 (CI 68–75) per 100,000 person-years in 2016. In women, the corresponding figures were 80 (CI 76–84) per 100,000 person-years in 1997 and 82 (CI 79–86) per 100,000 person-years in 2016.

During the 20-year study period, a total of 18,648 (27%) hardware removal procedures (52% women) were performed after primary ankle fracture surgery. The mean age at the time of the first hardware removal in 1997 was 53 years in women and 45 years in men. In 2016, the corresponding ages were 56 years in women and 57 years in men. The incidence of hardware removal procedures after ankle fracture surgery decreased from 31 (CI 29–32) per 100,000 person-years in the highest year, 2001 (n = 1,247), to 13 (CI 12–14) per 100,000 person-years in 2016 (n = 593) (Figure). In men, the incidence was 32 (CI 29–34) per 100,000 person-years in 2001 and 12 (CI 11–13) per 100,000 person-years in 2016. In women, the corresponding figures were 30 (CI 27–32) per 100,000 person-years in 2001 and 15 (CI 13–16) per 100,000 person-years in 2016.

The mean time between ankle fracture surgery and hardware removal procedure was 14 months. 43% of the hardware removals occurred during 0–3 months after surgery, 8% during 3–6 months, 15% during 7–12 months, 21% during 13–24 months, 5% during 25–36 months, and 8% at over 36 months (Table). Hardware removals were conducted within 3 months for 538 (52%) of the 1,029 patients who underwent hardware removal in the highest year, 2007 (Table). Thereafter, the removal rate remained steady until 2012 when the removal rate decreased markedly from 49% to 26% in 2016.

According to Finnish DRG-based hospital payment pricing, the direct costs for one ankle fracture surgery were €2,881 per patient in 2016. This amounted to an annual direct cost of ankle fracture surgery of approximately €9,500,000 in 2016. The DRG-based costs for one hardware removal are €797. Thus, the costs of removal procedures in 2016 were approximately €472,600, whereas the corresponding removal costs in 2001 were almost €994,000.

**Figure:**
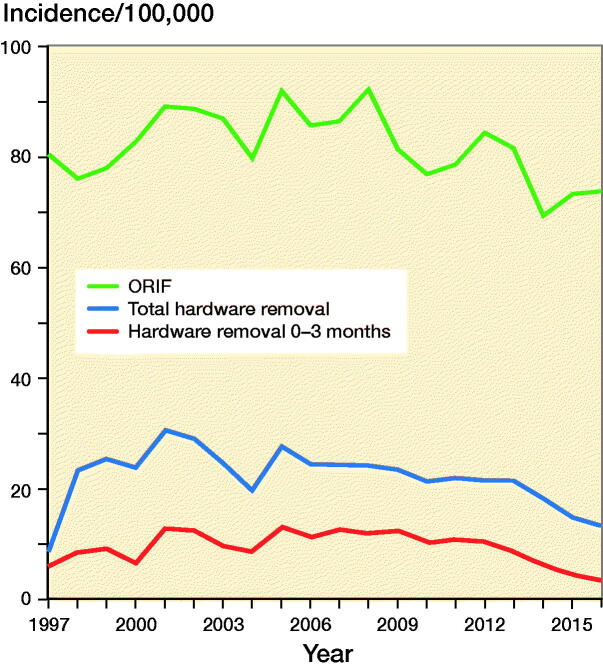
Incidence of ORIF, total hardware removals and hardware removals under 3 months in Finland between 1997 and 2016.

## Discussion

This study is the 1st nationwide study that shows a large number of ankle fracture surgeries (n = 68,865) as well as hardware removal procedures (27%; n = 18,648) during a 20-year study period. This study confirms the previous findings that the incidence of ankle fracture surgery is higher in young men and in older women (Kannus et al. 2016, Juto et al. 2018). The decreasing incidence of ankle fracture surgery might have been caused by the increasing knowledge of nonoperatively treated Weber-B type fractures, which can be either stable or unstable (Kortekangas et al. 2019). Stable ankle fractures can be treated non-surgically and account for about one half of all ankle fractures (Pakarinen et al. 2011, Van Schie-Van der Weert et al. 2012).

Previous studies have reported 12–80% of hardware removal (Richards et al. 1992; Sanderson et al. 1992, Bostman and Pihlajamaki 1996). This variation might be due to cultural and treatment policy differences and may also be attributed to the different lengths of observation periods or analytic methods between studies. The recent lowest hardware removal rates (13%) were reported by Fenelon et al. (2019). However, the authors suggest that their results were an underestimation because of the retrospective nature of their study that could have led to a larger loss to follow-up. Additionally, they showed that the majority of removals were due to symptomatic hardware. In Another retrospective study reported that about 17% of patients underwent hardware removal over a 3-year period (Naumann et al. 2016).

Our study showed that 27% of patients underwent hardware removal. A notable number, 8% (n = 1,516), of hardware removal procedures were performed after 3 years. Therefore, we believe that previous studies have underestimated the hardware removal rate due to shorter follow-up. Moreover, we assume that most of the removals performed after more than 3 years were due to symptomatic hardware. Williams et al. (2018) reported improvement in function following ankle implant removal, but their sample size was small (43 patients) and there was no control group. Patient symptomatic relief after hardware removal is still controversial.

Approximately 10% of all ankle fractures have concomitant syndesmotic injury. In 15–23% of operatively treated ankle fractures, a syndesmotic disruption necessitates surgical repair with a syndesmotic screw (Jensen et al. 1998, Egol et al. 2010). However, the need to remove this screw remains controversial. In his literature review in 2011 (including 7 studies between 2000 and 2010), Schepers (2011) reported that there is no need to routinely remove the syndesmotic screws. In a recent systematic review, Dingemans et al. (2016) also suggest that the current literature does not support the routine removal of syndesmotic screws. Furthermore, the complication rate for routine syndesmotic screw removal is about 20% (Schepers et al. 2011). Fenelon et al. (2019) showed in their study that 6% of all patients underwent planned hardware removal and that the majority of procedures were for the removal of a syndesmosis screw after a median time of 3 months. Our register study could not separate all hardware removals from only syndesmotic screw removal, but we assume that most removals within 3 months were of syndesmotic screws. This assumption is supported by the fact that these procedures decreased markedly between 2011 and 2016, most likely due to the changed evidence suggesting syndesmotic screws need not be removed (Figure) (Schepers 2011).

Hardware removal causes significant costs to patients, hospitals, and societies through the consumption of healthcare resources and absence from work. The total economic cost of removal is difficult to evaluate due to the multifactorial nature and financing of the Finnish healthcare system. However, Fenelon et al. (2019) reported the cost of hardware removal to be €1,113 per patient in Ireland. A study by Lalli et al. (2015) from the United States found the average cost of syndesmosis screw removal to be $3,579 ($287 to $9,981). In this figure, they included anesthesia, operating room and recovery room fees, as well as pharmacy, laboratory, and supplies costs. In our study, the DRG-based hospital payment system was used to reflect the cost of hardware removal (€794), but this sum does not include the costs of drug prescriptions, missed work, or loss of income.

According to our results, the annual cost of hardware removal decreased due to a decrease in removal rates (n = 1,247 in 2001 and n = 593 in 2016). In other words, in 2001 the cost of hardware removals was approximately €994,000 but in 2016 it was €472,600.

Bioabsorbable fixation materials have been suggested as a solution to the hardware removal problem. Good outcomes with bioabsorbable screws in ankle fractures without secondary surgery have been reported (Partio et al. 1992). In a meta-analysis including 4 studies comparing bioabsorbable and metallic screws, Wang et al. (2013) showed that all metallic screws were routinely removed 6–8 weeks after primary operation while only 2 symptomatic patients (3%) in the absorbable screw group needed re-surgery. In their meta-analysis, van der Eng et al. (2015) found no significant differences in the incidence of complications between patients treated with a polylactide acid (PLA)/polylevolactic acid (PLLA) screw and patients treated with a metallic syndesmotic screw. In the past, rapidly degrading polyglycolide acid (PGA) screws were associated with delayed inflammatory reactions, foreign body reaction, formation of a sinus, tract, or fistula, and osteolysis. However, there is no evidence that the PLA/PLLA combination has such problems (Bostman et al. 2005, Wang et al. 2013). The key advantage of using biomaterials is that hardware removal becomes unnecessary.

Our study has some limitations. Importantly, the study is register-based and bilateral operations on the same patient cannot be differentiated from the registry data. Additionally, the reason for removal remained unclear because the reason for removal is poorly recorded using ICD coding. Also, patient-related risk factors, such as smoking or alcohol consumption, are not recorded in the NHDR. A strength of the study is the accuracy and coverage of the NHDR database, which is collected from all Finnish hospitals. Indeed, the national coverage of the NHDR provided a large population of surgically treated ankle fractures for a 20-year period.

In summary, this 20-year nationwide study showed that 27% of patients underwent a hardware removal procedure after ankle fracture surgery. The number of routine syndesmosis screw removals seemed to decrease, resulting in lower economic costs. A substantial number of hardware removal procedures are being performed after the 3-year period.

All the authors made a substantial contribution to the conception of the study. NP planned the study and wrote the protocol. VM obtained permission from the Finnish National Hospital Discharge Register (FNHDR) and drafted and revised the article. TH planned the study, revised the protocol, and revised the article. HM planned the study and revised the article. All the authors critically revised the draft prepared by NP.

**Table 1.** Frequencies and ratios of under 3 months hardware removals by year

	Hardware removals		
Year	0–3 months	Total	Ratio (%)
1997	239	350	68
1998	343	941	37
1999	372	1,032	36
2000	269	971	28
2001	528	1,247	42
2002	511	1,195	43
2003	403	1,019	40
2004	364	820	44
2005	550	1,151	48
2006	475	1,028	46
2007	538	1,029	52
2008	512	1,034	50
2009	534	1,005	53
2010	446	924	48
2011	472	954	50
2012	456	941	49
2013	378	945	40
2014	267	807	33
2015	195	661	30
2016	152	593	26
